# Stochastic simulation of Boolean *rxncon* models: towards quantitative analysis of large signaling networks

**DOI:** 10.1186/s12918-015-0193-8

**Published:** 2015-08-11

**Authors:** Tomoya Mori, Max Flöttmann, Marcus Krantz, Tatsuya Akutsu, Edda Klipp

**Affiliations:** Bioinformatics Center, Institute for Chemical Research, Kyoto University, Gokasho, Uji, Kyoto 611-0011 Japan; Theoretical Biophysics, Humboldt-Universität zu Berlin, Invalidenstr. 42, 10115 Berlin, Germany

**Keywords:** Signal transduction, Systems biology, Probabilistic Boolean modeling, *rxncon*, Bipartite Boolean

## Abstract

**Background:**

Cellular decision-making is governed by molecular networks that are highly complex. An integrative understanding of these networks on a genome wide level is essential to understand cellular health and disease. In most cases however, such an understanding is beyond human comprehension and requires computational modeling. Mathematical modeling of biological networks at the level of biochemical details has hitherto relied on state transition models. These are typically based on enumeration of all relevant model states, and hence become very complex unless severely – and often arbitrarily – reduced. Furthermore, the parameters required for genome wide networks will remain underdetermined for the conceivable future. Alternatively, networks can be simulated by Boolean models, although these typically sacrifice molecular detail as well as distinction between different levels or modes of activity. However, the modeling community still lacks methods that can simulate genome scale networks on the level of biochemical reaction detail in a quantitative or semi quantitative manner.

**Results:**

Here, we present a probabilistic bipartite Boolean modeling method that addresses these issues. The method is based on the reaction-contingency formalism, and enables fast simulation of large networks. We demonstrate its scalability by applying it to the yeast mitogen-activated protein kinase (MAPK) network consisting of 140 proteins and 608 nodes.

**Conclusion:**

The probabilistic Boolean model can be generated and parameterized automatically from a *rxncon* network description, using only two global parameters, and its qualitative behavior is robust against order of magnitude variation in these parameters. Our method can hence be used to simulate the outcome of large signal transduction network reconstruction, with little or no overhead in model creation or parameterization.

## Background

Mathematical modeling of cellular regulatory networks is a challenge due to two opposite requirements: the aim to describe the biological complexity in all necessary detail and the need for simplicity that makes model analysis and simulation feasible.

Many signaling pathways and regulatory networks have been described with sets of ordinary differential equations (ODE). These models enable a representation of their general wiring and of the kinetics of individual reactions, and can be simulated to follow the dynamics of the investigated system (examples for the yeast MAPK pathways are, amongst many others, described in [1–7]). A frequently used framework to describe the dynamics of gene regulatory networks is Boolean modeling [8–10].

Both approaches helped to elucidate dynamic features of cellular networks, but both have their limitations. ODE models quickly become difficult to handle in larger networks. Boolean networks, on the contrary, are suitable to model larger networks because they simplify the potential values to binary ON or OFF, representing the activity or presence of compounds. This simplification enables us to describe and to analyze the dynamics of rather large networks, but neglects intermediate values, which may be of biological relevance.

When modeling signaling networks, the formation of protein-protein complexes and multiple phosphorylation or other modification steps quickly leads to a combinatorial explosion in the number of states. We have previously developed a method to cope with the biological complexity provided by the multitude of states of proteins, of interactions between proteins and dependencies restricting potential state changes. In the reaction-contingency (*rxncon*) formalism, all potential states and state transitions are listed together with the conditions (called contingencies) under which they can occur [11]. As a consequence, the state transitions are only executed if relevant conditions are met, i.e., when educts are present and contingencies are fulfilled. This description is similar to rule based models, and reduces the complexity drastically compared to a full ODE system [12]. The *rxncon* format is tailor made for formalization of biological knowledge from literature, and a *rxncon* based description can be used to automatically generate models that correspond to the network definition. With a recently presented bipartite Boolean model export, the resulting models can be simulated over time in a Boolean fashion [13].

In this paper, we extend this bipartite Boolean modeling formalism with probabilistic model export and simulation, as used in Probabilistic Boolean networks (PBN) [14]. PBNs are extensions of Boolean networks (BN), in which each node can have several update functions [15]. One of these is chosen randomly in each time-step according to the probabilities assigned beforehand, which makes state transitions non-deterministic. However, the model can also be interpreted quantitatively by averaging over a number of parallel realizations. Hence, this extension enables a semi-quantitative probabilistic simulation of regulatory networks in a bipartite PBN format.

Here, we use this concept to enable a quantitative probabilistic simulation of regulatory networks. It is based on the description in *rxncon* format and the Boolean model export and assigns probabilities to reactions. This way, it allows for a stochastic simulation of the reaction system and, hence also for a quantitative analysis. Using this method the contingency of a reaction on a modifier can be modeled as a certain probability *p* that the reaction depends on the modifier.

We benchmark the capabilities of the approach (i) with an example of an oscillatory system in the form of an isolated MAP kinase pathway with negative feedback, and (ii) by applying the method to the full MAPK network of *Saccharomyces cerevisiae* to investigate under which conditions the signal is reliably transmitted. Taken together, these examples demonstrate that the method scales with network size: It is largely insensitive to the assumption on parameter values, and can efficiently simulate large networks.

## Methods

### A network definition in the rxncon formalism uniquely defines a bipartite Boolean model

We previously defined the *rxncon* formalism for the representation of biological networks and described a way to generate Boolean models from it that can be simulated directly [13]. Briefly, the *rxncon* language describes a network in terms of decontextualized *elemental reactions*, their corresponding *elemental states*, and *contingencies* that define the contextual constraints on elemental reactions [11, 16]. An elemental state has a single molecular property defined, such as a specific phosphorylation of protein-protein interaction, and hence is a set containing all specific states that include this particular property. Elemental reactions define state transitions that produce or consume elemental states, and hence correspond to all specific reactions that change that particular property – regardless of the presence or absence of any other elemental state. Instead, such contextual conditions are provided by the contingencies, which define when reactions require or are enhanced by the existence or absence of other elemental states. Together, the elemental reactions and contingencies fully define the network, and can be directly exported to a bipartite Boolean model with a unique truth table [13].

The logic of the bipartite Boolean model is encoded in the rxncon language. Importantly, each reaction has a specific type that gives it certain properties (e.g., reversibility), but no parameters or kinetic laws are required. The reaction types include covalent modifications (e.g., phosphorylations (P+)), intra- and intermolecular interactions (e.g., protein-protein interactions (ppi)), production and consumption reactions (e.g., transcription (TRSC)) and translocation reactions (e.g., nuclear import (NIMP)). Which products a reaction creates depends on its type, e.g., a protein-protein-interaction of A and B (A_ppi_B) generates all complexes in which A binds to B (A--B). The contingencies determine if a reaction can take place given other states in the system (e.g., only if one of the reactants is phosphorylated on a specific residue). Contingencies can be absolute requirements, as denoted by an exclamation mark (!), absolute negative as denoted by an “×”, or quantitative contingencies that only express a quantitative influence which are denoted by K+/K-. The quantitative contingencies mean that the reaction rate switches between two non-zero levels. There are also contingencies that exclude any influence (0) or imply unknown connections (?), which are both treated as no effect.

**Fig. 1:**
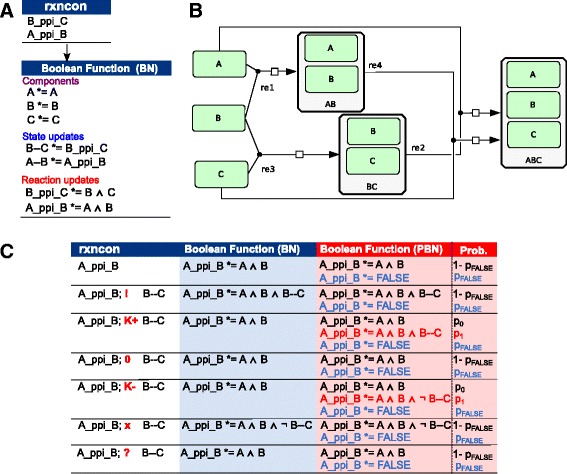
Principles of the *rxncon* formalism and the bipartite Boolean modeling. **a** Bipartite Boolean modeling based on *rxncon* format. Two protein-protein interactions between B and C (B_ppi_C), and between A and B (A_ppi_B), produce the B--C and A--B complexes, respectively. Note that these complexes are not mutually exclusive. Instead, they correspond to all complexes containing B & C and A & B. The trimeric A-B-C complex corresponds to the intersection between these two sets. The symbol “*=” means that the value of left side at time *t* + 1 will be equal to the value of right side at time *t*. **b** In a specific state description, these reactions translate into a network with three possible complexes (AB, BC and ABC) and four different reactions. Hence, the *elemental* reactions in (**a**) are generic statements that map to several distinct *specific* reactions in (**b**). If the specific reactions corresponding to a single elemental reaction occur with different rates, then the elemental reaction is contingent on one or more of elemental states. There are six such possible contingencies (**c**); absolute requirement (A_ppi_B; ! B--C)(corresponding to rate k_re1 = 0 in (B)); positive effect (*K*+)(k_re1 < k_re2 in (B)); neutral (0)(k_re1 = k_re2 in (B)); negative (*K*-)(k_re1 > k_re2 in (B)) and absolutely inhibitory (x)(k_re2 = 0 in (B)). Unknown effects (?) are treated as neutral. In a qualitative Boolean model, only absolute requirements or inhibitions affect the update functions, while the probabilistic model can account also for the quantitative modifiers (*K*+/*K*-), as can be seen in the direct comparison between the two model formats **c**: In this table, protein-protein interaction B_ppi_C is omitted. The false rules (blue lines in red box) correspond to noise in biological systems, and can be set to any value between 0 (no noise) and 1 (no signal). The second Boolean functions in the rows “A_ppi_B; K+ B--C” and “A_ppi_B; K- B--C” (red lines) only appear in the probabilistic model, allowing it to account for quantitative modifiers (*K*+/*K*-), which was not possible with the qualitative version. In the probabilistic Boolean modeling, one of the Boolean functions is chosen randomly at each time step according to the probability assigned to it

Given such a network, we can derive a BN that can be used to simulate the network dynamics using a set of rules. The resulting BN has a bipartite structure split into reaction nodes and state nodes (Fig. 1a). The update function for a reaction depends on its substrates and its contingencies while the update function for a state depends on the reactions connected to it. The product states of non-reversible reactions require active degradation, and are only set to FALSE if there is an active consumption reaction and no producing reaction. Hence, these states need to have a memory, which is implemented as self-dependence in the model. Reversible reactions must be TRUE for their product states to stay TRUE, and their products are assumed to degrade when they are FALSE. We give a short example in *rxncon* format

B_ppi_C

A_ppi_B; ! B--C,

where “ppi” is a protein-protein-interaction and A, B, and C are protein components of the system. Following this definition, B and C can always interact to form the complex “B--C” which in turn enables the interaction between A and B if present. It is important to stress that the complex B--C is not a specific state, but rather the entire set of states that includes B bound to C (in this case, both the BC only complex, and the ABC complex; Fig. 1b). The resulting Boolean network from this definition would be:

$$ \begin{array}{l}B--C\left(t+1\right)=B\_ppi\_C(t)\\ {}A--B\left(t+1\right)=A\_ppi\_B(t)\\ {}A\_ppi\_B\left(t+1\right)=A(t)\wedge B(t)\wedge B--C(t)\\ {}B\_ppi\_C\left(t+1\right)=B(t)\wedge C(t)\end{array} $$

with *t* being the current time-step. So we get an update function for the two possible states and their producing reactions.

If a *rxncon* definition contains a reaction that degrades or synthesizes a component, we need to add more logic to the update functions to ensure that a state is only TRUE if these functions are in the right configuration. Essentially, states require their components to be there. Hence, if a component belonging to a state is degraded and not synthesized at the same time it cannot be TRUE, regardless of any other reactions.

The differences in the update functions for other types of contingencies are shown in Fig. 1c. As shown there, non-absolute contingencies that represent a gradual influence of a state on a reaction are simply ignored in this approach, which is a strong limitation for the results. We address this shortcoming using the probabilistic approach presented here.

### Extension towards probabilistic simulations enables the use of quantitative modifiers

In the Probabilistic Boolean network approach each node *×* can have more than one update function and each of the update functions *f*_*i*_ has an assigned probability *p*_*i*_ to be chosen in each time-step. We use a synchronous updating scheme and an instantaneously random probabilistic Boolean network (all functions can be chosen in all time-points) without changes to the probabilities over time and thus produce a Markov chain. To be able to represent quantitative contingencies in Boolean simulations, we assign each reaction that depends on a quantitative contingency at least two update-functions; one depending on the contingency and one that does not (Fig. 1c). We use the functions' probabilities as parameters for how strong the reaction relies on the contingency, so that a reaction depends on a contingency only with the probability *p*_*i*_ and is independent with the probability 1-*p*_*i*_.

To be able to model a small random failure of the whole reaction and test the models robustness we add one update function that always evaluates to FALSE and which has a probability of *p*_*FALSE*._

**Fig. 2:**
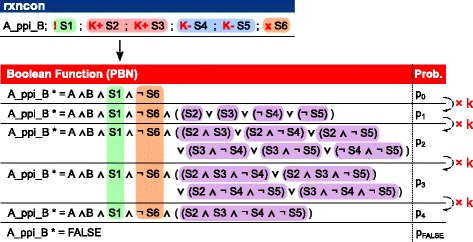
The probabilistic bipartite Boolean modeling method can handle complex cases. Here, we consider a reaction with one absolute requirement (!S1), two positive quantitative modifiers (K+ S2, K+ S3), two negative quantitative modifiers (K- S4, K- S5) and one absolute inhibition (× S6). The Boolean functions are generated according to the number of positive (red box) that are TRUE and negative effectors (blue box) that are FALSE. The probability assigned to each Boolean function, except for the false function, increases *k* times as the number of fulfilled quantitative conditions in a set (purple box) increases, where the total probability of the Boolean functions (= *p*_0_ + *p*_1_ + *p*_2_ + *p*_3_ + *p*_4_ + *p*_FALSE_) is 1

For complicated cases (such as multiple modifiers affecting one reaction) multiple Boolean functions are generated depending on the number of positive and negative effectors and different probabilities are assigned to them (Fig. 2). Let *p*_0_ and *p*_*FALSE*_ be the probabilities assigned to the Boolean function without contingencies and the false function, respectively, then *p*_0_ is given by the following equation:

$$ {p}_0=\left(1-{p}_{FALSE}\right)/{\displaystyle {\sum}_{i=0}^n{k}^i,} $$

where *n* is the total number of positive and negative quantitative modifiers. The parameter *k* scales the probability differences between the update functions so that each probability *p*_*i*_ is *k* times larger than the probability *p*_*i*-1_. The *i*-th Boolean function is assigned a probability of *p*_*i*_ = *p*_0_ · *k*^*i*^. For *k* > 1, this scaling guarantees higher probabilities for more restrictive update functions (Fig. 2), because these are ordered by stringency. In each update function *f*_*i*_ (1 ≤ *i* ≤ *n*), we include the conjunction of *i* contingencies. For each *f*_*i*_ this leads to $$ \left(\begin{array}{c}\hfill n\hfill \\ {}\hfill i\hfill \end{array}\right) $$ possible combinations of contingencies. We require at least one of these combinations to be TRUE by taking their disjunction. The update functions become more restrictive for larger *i* by requiring at least *i*. modifiers to be TRUE, meaning that if *f*_*i*_ = *TRUE* it follows that *f*_*i*-1_ = *TRUE*. In summary, this means that each update function *f*_*i*_ requires at least *i* contingencies to be TRUE and has a probability to be chosen that is proportional to *k*^*i*^. If we consider the simple example above and add different quantitative contingencies

B_ppi_C

A_ppi_D

A_ppi_B; K+ B--C; K- A--D

we get to the following update functions for the reactions following the rules above with the parameters *k* = 10 and *p*_*FALSE*_ = 0.1:

$$ \begin{array}{cccc}\hfill A\_ppi\_D\left(t+1\right)\hfill & \hfill =\hfill & \hfill A\wedge D(t)\hfill & \hfill 0.9\hfill \\ {}\hfill A\_ppi\_D\left(t+1\right)\hfill & \hfill =\hfill & \hfill 0\hfill & \hfill 0.1\hfill \\ {}\hfill A\_ppi\_B\left(t+1\right)\hfill & \hfill =\hfill & \hfill A(t)\wedge B(t)\hfill & \hfill 0.008108\hfill \\ {}\hfill A\_ppi\_B\left(t+1\right)\hfill & \hfill =\hfill & \hfill A(t)\wedge B(t)\wedge \left(B--C(t)\vee \neg A--D(t)\right)\hfill & \hfill 0.08108\hfill \\ {}\hfill A\_ppi\_B\left(t+1\right)\hfill & \hfill =\hfill & \hfill A(t)\wedge B(t)\wedge \left(B--C(t)\wedge \neg A--D(t)\right)\hfill & \hfill 0.810812\hfill \\ {}\hfill A\_ppi\_B\left(t+1\right)\hfill & \hfill =\hfill & \hfill 0\hfill & \hfill 0.1\hfill \\ {}\hfill B\_ppi\_C\left(t+1\right)\hfill & \hfill =\hfill & \hfill B(t)\wedge C(t)\hfill & \hfill 0.9\hfill \\ {}\hfill B\_ppi\_C\left(t+1\right)\hfill & \hfill =\hfill & \hfill 0\hfill & \hfill 0.1\hfill \end{array} $$

The number of Boolean update functions for a reaction with *n* quantitative modifiers is *n* + 2 as we iterate the number of required modifiers from 0 to n, plus the *p*_FALSE_ function. It is worthy to note that we only need two parameters (*p*_FALSE_ and *k*) to generate a simulation-ready model, but the user is able to change each of the update probabilities to their needs in the output file.

### Implementation

The *rxncon* tool is published as open source software (under lGPL). The tool as well as its source code can be downloaded freely from http://www.rxncon.org. The standard Boolean model is generated in BooleanNet format (https://github.com/ialbert/booleannet) and simulated in BooleanNet [17]. Since BooleanNet does not provide probabilistic Boolean simulation, we added the capability to simulate probabilistic Boolean models using BoolNet [18], an R (http://www.r-project.org) package for probabilistic Boolean networks. Accordingly, the *rxncon* tool was modified such that it can create a model in the BoolNet format.

The *rxncon* tool is implemented in Python and Javascript. Here, we added the probabilistic model generation to the *rxncon* tool (http://www.rxncon.org). The tool can be used online and we provide a desktop version for all major platforms as a download.

## Results and discussion

In the Methods section, we introduced the concept of probabilistic simulation of a *rxncon*-derived Boolean network and assigned probabilities to execute reactions according to their rules or to contradict them (false-rate). Below, we exemplify the approach for selected signaling networks in order to analyze the effect of assigning false-rates and a k-base for combining probabilities for multiple contingencies. We illustrate the effect of parameter choices on the oscillatory behavior of a pathway with negative feedback. Finally, we demonstrate that the approach can be applied to a large signaling network.

### Application: oscillatory system representing simplified signaling pathway with negative feedback

**Fig. 3:**
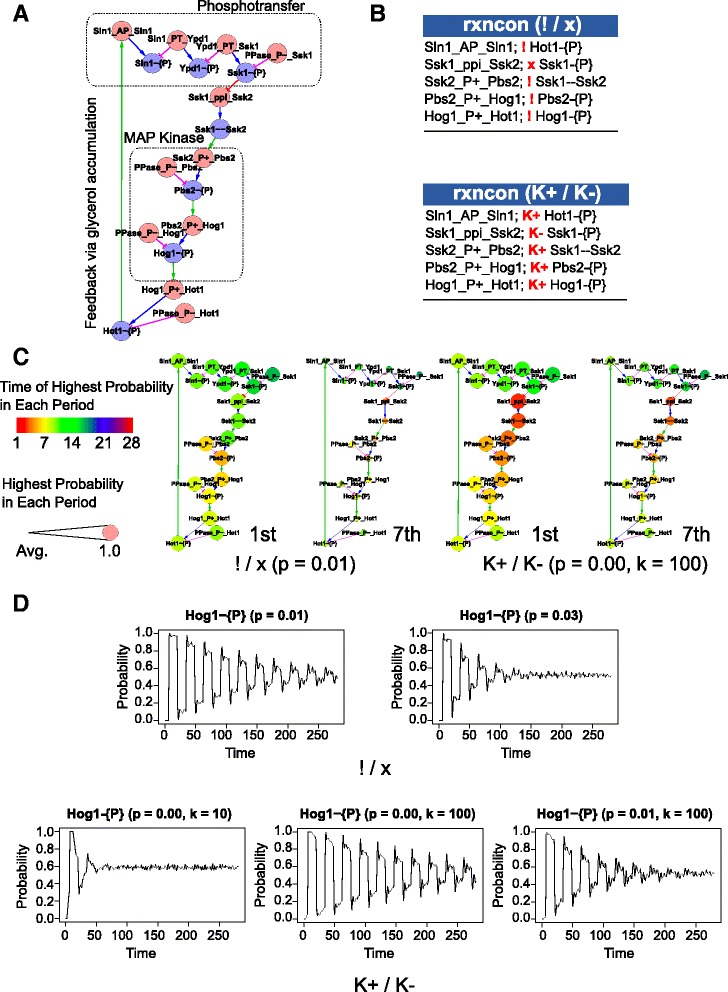
The probabilistic method can simulate dampening of oscillations in a linear signaling pathway with negative feedback. **a** Simplified model of the high osmolarity glycerol (HOG) pathway visualized as a regulatory graph [11]. The simplified HOG pathway consists of two modules: a phosphotransfer module and a MAP kinase module [13]. When turgor is sufficient, the phosphotransfer module is active and keeps the downstream MAP kinase module inactive. On the other hand, increased external osmolarity causes loss of turgor, which leads to inactivation of the phosphotransfer module and activation of MAP kinase module. The output of the signal cascade activates the phosphotransfer module again via glycerol accumulation, which leads to turgor recovery. **b** Descriptions of the simplified HOG pathway in the *rxncon* format. The upper panel displays the qualitative HOG model, and the lower panel the quantitative HOG pathway in which all contingency symbols were changed from “!” or “×” into “*K*+” or “*K*-”, respectively, indicating a certain probability of the occurrence of a reaction if its contingencies are met or not met. **c**, **d** Results of the time course simulation of the qualitative HOG model with a non-zero failure rate (*p*_FALSE_ > 0) and the quantitative HOG model. The average probabilities of being active or inactive are calculated over 1000 time series of 280 time points. **c** The amplitude and phase for the 1st and 7th periods are represented by the size and colors of nodes, respectively. The parameters *p* and *k* shown in each panel indicate false-rate and k-base value, respectively. **d** Individual state transitions of Hog1-{P} with different probabilities of false-rate and different scale factors of k-base. The upper (“!/×”) and lower (“*K*+ / *K*-”) panels show the average value for the phosphorylated state of the terminal MAP kinase (Hog1-{P}) in the qualitative HOG model and the quantitative HOG model, respectively

To show the capabilities of our probabilistic approach, we used the simplified high osmolarity glycerol (HOG) pathway of *S. cerevisiae* as an example of oscillatory system (Fig. 3a) [13]. The simplified HOG pathway consists of two modules: a phosphotransfer module and a MAP kinase module. A signal from an external module leads to inactivation of the phosphotransfer module and activation of the MAP kinase module, and then the output of the signal cascade in the MAP kinase module feeds back to the phosphotransfer module *via* glycerol accumulation (reviewed in [19]). The *rxncon* definitions of the pathway are illustrated in Fig. 3b and implemented as a qualitative (top) or quantitative model (bottom).

We analyzed time series of the qualitative and quantitative HOG pathway with fixed start states. To be able to follow the dynamics of the network, we simulated 1000 runs of the probabilistic model and averaged over the Boolean state of each node in all simulations to calculate a probability of activation. In a standard simulation only the network nodes that were defined as components in the *rxncon* system (the basic proteins in this case) were set to TRUE in the start state. The simulation results are shown in Fig. 3c and d. They reveal that high false-rates and low k-base values lead to a fast convergence of the system to a steady state (of about 0.5) after averaging over 1000 simulations. However, what appears to be dampened oscillations is an effect of a loss of synchrony of oscillations between the single simulation runs. For the qualitative model (without k values), when false-rate was 0.01, the system converged gradually to a state around 0.5 (Fig. 3c left, Fig. 3d upper panels). On the other hand, when the false-rate was 0.03, oscillations broke down early because of rapid desynchronization. Compared to that, synchronous oscillations were kept for a long time when k-base value was high. This is because the model with high k-base values well approximates the qualitative model. Finally, we used a low false-rate (*p*_FALSE_ = 0.01) and a high k-base value (*k* = 100) and generated time series. In this case, oscillations were kept in early stage and the system converged gradually (again to about 0.5). These results revealed that our approach, with appropriate false-rates and k-base values, enables more realistic Boolean simulation of biological systems (Fig. 3c right, Fig. 3d lower).

### Scalability: the method can be used to simulate the entire MAP kinase system

We applied our probabilistic approach to MAP kinase network of baker’s yeast as an example of realistic signaling networks. The MAP kinase network is related to control of cellular functions such as stress response. As described in [11], the first *rxncon* model of the MAP kinase network was constructed based on literature, and contains 84 components, 181 states, and 222 reactions. We later translated this network into a qualitative bipartite Boolean model, in which all contingencies are absolute, and made minor adjustments because the bipartite Boolean modeling approach cannot deal with quantitative models [13]. This work also extended the network model to encompass 142 components, 182 elemental states, and 273 elemental reactions, in order to make the pathways functional in the model. We used this previously published network and further modified it to a quantitative model by reverting to the original quantitative contingencies [11].

**Fig. 4:**
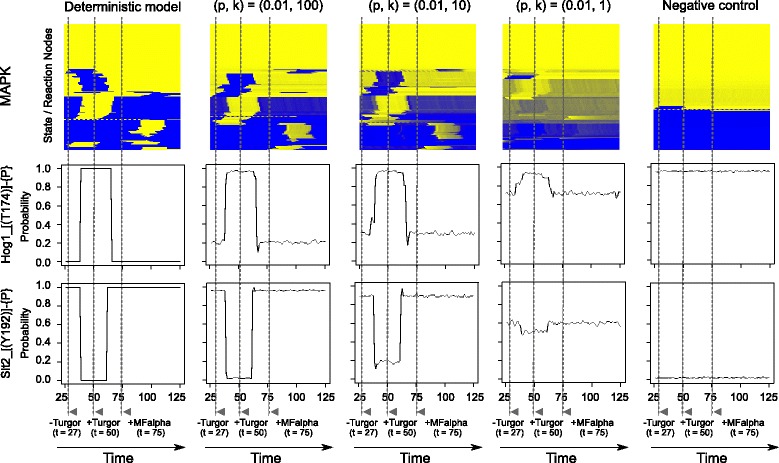
The probabilistic method scales efficiently, and can be used to simulate the entire yeast MAP kinase network. The complete MAP kinase network [11] was used to generate models with variable k-base values. The top panels show the state evolution of all model species in the MAP kinase network model as a heat map. The average probabilities of active or inactive are calculated over 1000 time series of 125 time points. As the average probability increases, the color changes from blue (false) to yellow (true) gradually. *p* and *k* indicate false-rate and k-base, respectively. The initial setting was (turgor, MFalpha, Ste3, Tec1) = (true, false, false, true), but turgor was turned off at time *t* = 27, then switched on again at time *t* = 50 [13]. MFalpha was added at time *t* = 75. The individual state transitions of Hog1_[(T174)]-{P} and Slt2_[(Y192)]-{P} are shown in the middle and bottom panels. The rightmost panel shows a negative control in which all “*K*+” and “*K*-”contingencies were deleted

State evolution of the MAP kinase network and individual state curves of phosphorylated Hog1 and Slt2 with varying k-base are shown in Fig. 4. This comparison shows the effect of different parameter sets on the output signal of the network. The deterministic simulation uses absolute contingencies without false-rates as shown in [11]. As a negative control, we used a modified version of the same MAP kinase network model in which the effects of quantitative modifiers was not taken into account (i.e., all contingencies “*K*+/*K*-” were erased). This modification completely removes the effect of the quantitative modifiers in the network, and destroys the information transfer ability of the pathways that no longer respond to perturbations. In these simulations, we ran the model to steady state where we turned off turgor at time *t* = 27, switched it on again at time *t* = 50, and then turned on MFalpha at time *t* = 75 in order to analyze the cross-talk effects. The simulation results show that the state evolution gets smoother as the k-base value becomes smaller, while the system becomes noisier. We explored a range from almost deterministic simulation (*k* = 100) to equal chances of choosing rules with no bias depending on the number of fulfilled contingencies (*k* = 1). However, the signal can be seen even when the *k*-base value is 1, in spite of very high background. This indicates that the system is quite robust against change of k-base value.

## Conclusion

### The probabilistic approach enables quantitative analysis of rxncon-derived Boolean networks

The presented extension of the Boolean approach to a probabilistic Boolean network allows for a stochastic simulation of regulatory and signaling networks, which are already presented in the *rxncon* formalism. It can, hence, respect important biological details and contingencies, but also include probabilities for the occurrence of reactions.

We have demonstrated the impact of the probabilistic simulation for a small example and a large realistic MAP Kinase network. It can be noticed that the non-deterministic simulation can exhibit system properties that will not occur in a deterministic simulation. For the isolated HOG pathway with artificially introduced negative feedback we observed de-synchronization and hence a dampening of the oscillations in the simulated ensemble, while the oscillations are stable in the deterministic system. The full MAPK system showed, on the ensemble level, that a visually detectable signal is transmitted even with very low k-values, which could be interpreted in the sense of robustness of the signaling network.

To get statistically meaningful results in stochastic simulations one usually averages over large numbers of replicates, as we have also done here. This enabled to see in the MAPK network visually distinguishable signals in the pathway output even with no bias towards choosing rate laws with fulfilled contingencies (k-base =1). There are various ways to interpret these results and it is common to regard each iteration as the pathway output in one cell and consider the result as a population mean. However, the iterations could also be interpreted as isolated instances of signaling pathways in one cell, in which case multiple parallel pathways compensate for high noise levels. Such pathways would need to be insulated from each other (e.g., by scaffolding as is known for the Hog pathway (Pbs2; [20])).

Depending on this interpretation, the obtained probabilities in pathway output correspond to a quantitative simulation of either the behavior of many pathways in one cell or an average over many cells. While this modeling strategy cannot compare to the precision of ODE models, it provides a much closer approximation than classical or binary reaction-contingency based Boolean modeling.

### The probabilistic Boolean approach based on rxncon is automated and can be reused

Model generation and parameterization is generally a major challenge, even given a reasonable base of knowledge about the system. Typically, structuring and parameterization require a large efforts and even Boolean models need decisions on exact truth tables. The challenge is even more daunting in quantitative models. This has been tackled in approaches such as ODE-fy [21] or SQUAD [22], where at least threshold levels or kinetic parameters such as Hill coefficients or Km values must be set. In ODE models in general, parameter estimation is an art even given a fully determined model structure [23]. Here, using a special case of PBN modeling, we parameterize a full MAPK network model using only two parameters, and show that the qualitative outcome is robust against large changes in both. Hence, the proposed method supports full automation of model generation from a biological knowledge database (in the *rxncon* format). Given sufficient suitable experimental data, however, further more detailed parameterization is possible.

The direct link to an underlying database is specifically useful in the light of network reconstruction and curation in combination with model quality assurance, which is a time consuming work requiring knowledge and effort of experts [24, 25]. The fact that models can be easily reused is of great importance for an ongoing description of biological network [16]. Here, it is exemplified for networks of the yeast *S. cerevisiae*, but would be of even greater importance for human signaling networks relevant in health and disease. Our approach is in line with standardization efforts as supported by SBML and it enables model annotation. The method offered here has the advantage that it is based on a database format that allows high level of annotation, and that can be used for other exports, i.e., into SBML or various visualization tools, such as Cytoscape. Hence, a model developed in this format can also serve as a database and can easily be reused and extended in other contexts.

### The framework allows for scalable quantitative modeling

Perhaps the premier challenge for formulating appropriate mathematical models is the complexity of signaling networks, especially due to formation of multiple complexes and many different post-translation protein modifications. While methods for compact model definition have been developed [12], the computational demand on simulating large complex networks is daunting. e.g., the yeast pheromone model contains a single MAP kinase pathway with 19 species, but the reaction rules correspond to over 200,000 specific states (see yeastpheromonemodel.org). Here, we model the complete MAP kinase network consisting of a much larger number of components. Despite the large network size, both model generation and simulation are quick (simulation takes only about 10 s on a standard PC). The main advantage with the reaction-contingency based model structure is that it avoids the combinatorial explosion of the possible interactions and modifications, hence models scale very nicely with the number of components and reactions, and the method will be computationally efficient also for much larger models.

## Abbreviations

BN: Boolean networks
HOG: High osmolarity glycerol pathway
MAPK: Mitogen-activated protein kinase
ODE: Ordinary differential equations
PBN: Probabilistic Boolean networks
